# RNA-seq Based Transcription Characterization of Fusion Breakpoints as a Potential Estimator for Its Oncogenic Potential

**DOI:** 10.1155/2017/9829175

**Published:** 2017-10-17

**Authors:** Jian-lei Gu, Morris Chukhman, Yao Lu, Cong Liu, Shi-yi Liu, Hui Lu

**Affiliations:** ^1^Shanghai Institute of Medical Genetics, Shanghai Children's Hospital, Shanghai Jiao Tong University, Shanghai 200040, China; ^2^Department of Bioinformatics, SJTU-Yale Joint Center for Biostatistics, Shanghai Jiao Tong University, Shanghai 200240, China; ^3^Key Laboratory of Molecular Embryology, Ministry of Health and Shanghai Key Laboratory of Embryo and Reproduction Engineering, Shanghai 200040, China; ^4^Department of Bioengineering, Bioinformatics Program, University of Illinois at Chicago, Chicago, IL 60607, USA

## Abstract

Based on high-throughput sequencing technology, the detection of gene fusions is no longer a big challenge but estimating the oncogenic potential of fusion genes remains challenging. Recent studies successfully applied machine learning methods and gene structural and functional features of fusion mutation to predict their oncogenic potentials. However, the transcription characterizations features of fusion genes have not yet been studied. In this study, based on the clonal evolution theory, we hypothesized that a fusion gene is more likely to be an oncogenic genomic alteration, if the neoplastic cells harboring this fusion mutation have larger clonal size than other neoplastic cells in a tumor. We proposed a novel method, called iFCR (internal Fusion Clone Ratio), given an estimation of oncogenic potential for fusion mutations. We have evaluated the iFCR method in three public cancer transcriptome sequencing datasets; the results demonstrated that the fusion mutations occurring in tumor samples have higher internal fusion clone ratio than normal samples. And the most frequent prostate cancer fusion mutation, TMPRSS2-ERG, appears to have a remarkably higher iFCR value in all three independent patients. The preliminary results suggest that the internal fusion clone ratio might potentially advantage current fusion mutation oncogenic potential prediction methods.

## 1. Introduction

Chromosomal rearrangement events often lead to gene fusion mutation and result in a hybrid fusion gene, consisting of two separate fusion parents (genes) [1, 2]. Gene fusion is an important class of genetic alterations in human cancers; it causes about 20% of human cancers [3]. In the last decades, a large number of important fusion mutations have been recognized [3], including the first identified “Philadelphia chromosome” BCR-ABL gene fusion in chronic myelogenous leukemia [4], the important biomarker of synovial sarcomas, SYT–SSX gene fusion [5], and the most studied fusion TMPRSS2-ERG in prostate cancer [6]. However, distinguishing oncogenic fusion mutations, whose functions are critical for cancer initiation, progression, and metastasis, remains a big challenge. Traditionally, a fusion event is considered as an oncogenic mutation if it occurs more frequently in cancer patients (i.e., high recurrent rate) [2, 7]. However, this strategy is expensive and time-consuming to conduct experiments for many patients. Moreover, this method has limited power to predict the oncogenic potential of novel and rare fusion mutations for a certain patient, and thus its application in the era of precise medicine is limited.

Currently, several studies have attempted to predict the oncogenic potential for fusion mutations. Shugay et al. implemented 24 structural and functional features of known oncogenic fusion genes and then predict the oncogenic potential for novel fusion genes by a SVM (Support Vector Machine) classifier [8]. Wang et al. developed an algorithm to nominate biologically important fusion mutations by integrating various molecular interactions, pathways, and functional annotations [9]. Wu and his colleagues used a molecular network based method to prioritize oncogenic fusion genes [10]. These machine learning based methods all relied on sequence structural and functional features of fusion genes. However, due to the incompleteness of included features under investigation, these methods could be biased. Moreover, the transcription characterizations of fusion genes were ignored by these methods.

It is widely accepted that tumor has heterogeneous cell composition, which can be viewed from Darwin's evolutionary perspective as a heterogeneous population of neoplastic cells [11]. The mutation-endowed genetic alteration in cancer reflects the “survival” fitness of neoplastic cells. The neoplastic clones harboring “driver” mutations could be expanded during the progression of cancers. Thus the dynamic changes of specific clonal size also might reflect the oncogenic potentials of specific mutations [11, 12]. Based on this concept, we hypothesized that a fusion gene is more likely to be an oncogenic mutation if the neoplastic subclone harboring this fusion mutation has a larger population size, compared to other clones. And if we could estimate the clonal size of neoplastic cells, harboring a certain fusion gene, it might be helpful to predict the oncogenic potential of fusion mutations in tumor sample.

To achieve this goal, there are two fundamental questions that need to be answered: (1) if there is only transcriptome sequencing data, how can we estimate the relative subclone size in a mixture tumor sample? (2) Does this estimator have enough power to distinguish “oncogenic” fusion genes from “passenger” background? The best way to infer detecting subclonal heterogeneity is to analyze somatic DNA alterations by exome or genomic sequencing. However, if we only have RNA-seq data available, we proposed a new transcript-based method, named iFCR, to estimate the relative subclone size of neoplastic cells, harboring a certain fusion mutation. Public glioblastoma single-cell sequencing data was used to test this assumption. To address the second problem, we applied iFCR to two public datasets, including a breast cancer cell line dataset and a primary prostate tumors (with adjacent normal tissues) dataset, where the breast cancer cell lines, with homogeneous cell compositions, was used to simulate the early-stage “oncogenic” fusion mutations in primary tumor samples. In the following context, we will describe this new method in detail and then demonstrate the results of applying this estimator to two datasets.

## 2. Results

### 2.1. The Estimation of Relative Clone Size by iFCR

**Figure 1:**
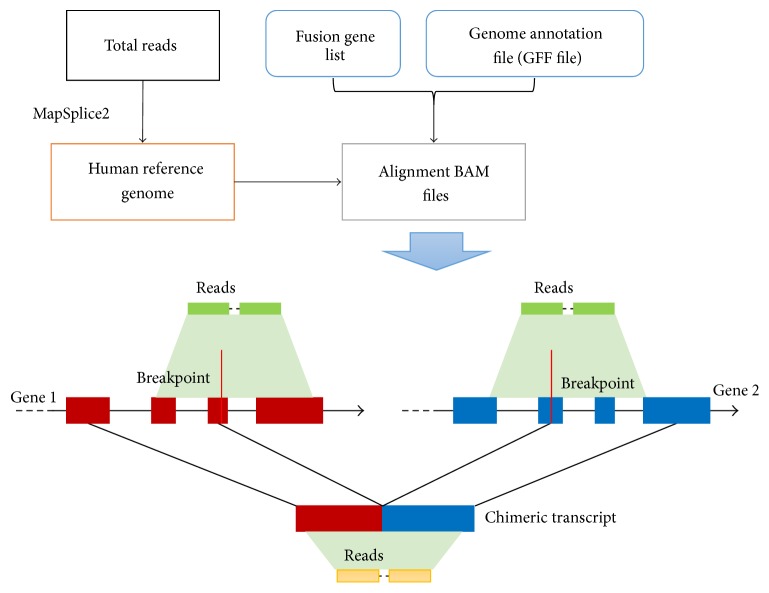
The diagram of realignment procedure to identify the overlapping reads of parents' transcripts. The RNA-seq data realigned to the corresponding reference genome, and the genome annotation file (GTF) and fusion mutations were used to retrieve these overlapping reads of parent genes. The red boxes represent the exonic sequences from Gene 1 and blue boxes are from Gene 2. The sequencing reads aligned onto the breakpoint of Gene 1 and Gene 2 were called overlapping reads; the sequencing reads aligned onto the breakpoint of chimeric transcript were called fusion reads. Breakpoints could occur in exonic region, intronic region, and UTR region.

Traditionally, the reconstruction of subclone structure is based on in situ hybridization method [13, 14] or DNA sequencing technology [15, 16]. However, gene fusion studies used transcriptome sequencing technology and merely accompanied genome sequencing data in the same sample. In order to estimate the subclone structure based on transcriptome sequencing data, we make a simple assumption that fusion genes and their parent genes have similar expression level among neoplastic cells in the same sample. Based on this assumption, the proportion of subclone size could be represented as the ratio of expression level between chimeric transcripts and their corresponding normal parent's transcripts. This ratio, defined as iFCR, reflects the subclone proportion of specific chimeric subclones in the heterogeneous neoplastic cells. However, as Figure 1 shows, a gene fusion mutation is the juxtaposition of two separate genes; the breakpoint region is the only different part between chimeric transcript and their parents' transcripts. To represent the relative quantities of chimeric transcripts, the sequencing reads that aligned onto the breakpoint of a chimeric transcript and represented the number of chimeric transcripts were called fusion reads in this study. Correspondingly, the sequencing reads that aligned onto the breakpoint of their parents' transcripts and represented the number of normal parents' transcripts were called overlapping reads. In this work, we directly used the number of fusion reads from original published articles, and a realignment procedure was designed and performed to retrieve these overlapping reads. The details of this procedure are described in Methods.

To test this method, we used a single-cell sequencing study of glioblastoma dataset (SRP042161) and detected the fusion mutations of each single-cell sequencing library for each tumor sample. So we calculated the heterogeneity of fusion clones in two ways: (1) by summing the reads supporting the fusions and their parents in of the single-cells, we were able to calculate their iFCR^average^ value. (2) As another calculation, for each tumor sample, we counted the number of cells each fusion was identified in and the number of cells that the parent genes in that fusion had nonzero transcript counts in and calculated a “real” ratio of the number of fusion clones and normal clones that is calculated from the cell counts rather than the transcripts counts. As Figure 3 shows, the log of the iFCR value linearly correlated with the “real” ratio of number of fusion cells and normal cells.

**Figure 2:**
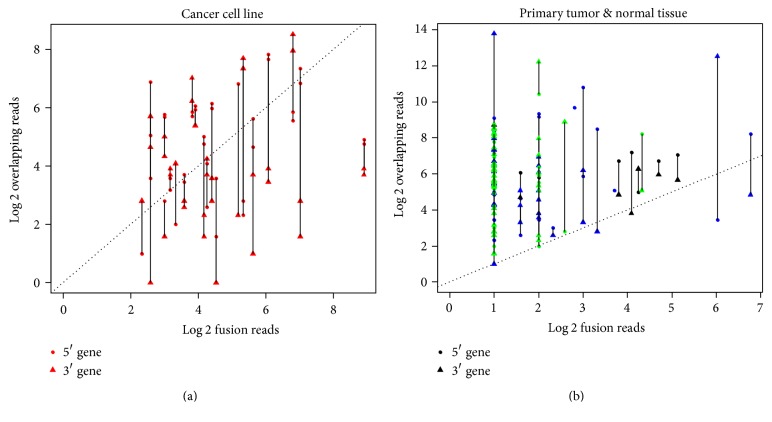
The dot-scatter plot comparison of the number of fusion reads and the overlapping reads. Diagram (a) shows fusion mutations in cell line samples. Diagram (b) shows fusion mutations in primary tumor and normal tissue samples. The green, blue, and black dots in diagram (b) represent normal counterparts, tumor, and recurrent fusion mutations, respectively. Dots give an observational estimate that fusion mutations occurring in tumors and normal tissues have more normal overlapping reads than in cancer cell lines.

**Figure 3:**
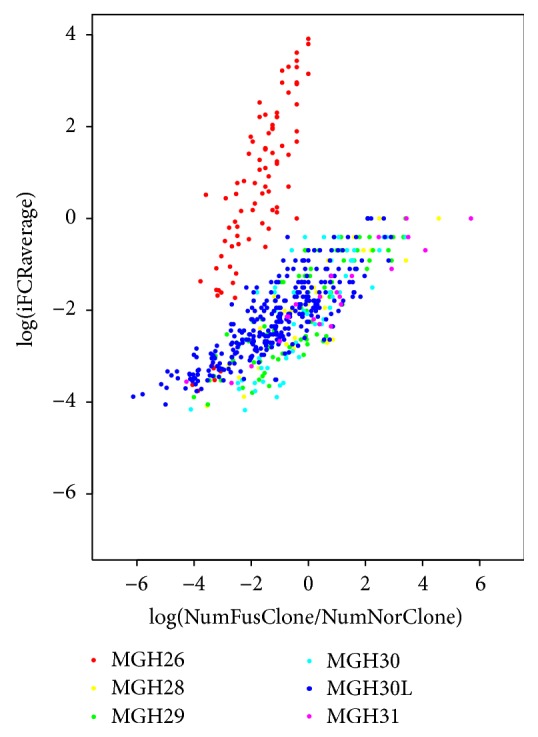
The logged iFCR value linearly correlated with the “real” ratio of number of fusion cells and normal cells. The* x*-axis is the ratio of number of fusion cells and normal cells for a certain fusion mutation. The* y*-axis is the iFCR^average^ value for a certain fusion mutation. Each dot represents the iFCR value and the ratio of number of fusion cells and normal cells for a certain fusion mutation. The dots with different colors represented the different sequencing libraries from 5 individual tumor samples (MGH26, MGH28, MGH29, MGH30, and MGH31). MGH31L is sequenced by long reads (100 bp).

Theoretically, breast cancer cell lines, consisting of homogeneous cells, should have lower heterogeneity, while higher heterogeneity should be expected in primary tumors and their adjacent normal tissue. To evaluate whether iFCR could capture this pattern, we compared the read distributions between two datasets. There are 62% (25/40) and 54% (20/37) chimeric transcripts that have reads mapped to full-length transcripts of both parents' genes in prostate tumors and normal, respectively. This proportion reduced to 30% (7/23) in breast cancer cell lines. As Figure 2 shows, in most breakpoints of breast cancer cell lines, more reads could be mapped to chimeric transcripts (i.e., fusion reads) than parents' genes (i.e., overlapped reads), while this ratio is reversed in primary prostate tumors and their adjacent normal tissues. Specifically, in adjacent normal tissues (green), all the chimeric transcripts carry less reads in chimeric transcripts than those in parents' genes. These results suggest iFCR might be a useful ratio in estimating tumor heterogeneity.

### 2.2. iFCR Distribution Is Correlated with Recurrent Rate in the Prostate Tumor Dataset

**Figure 4:**
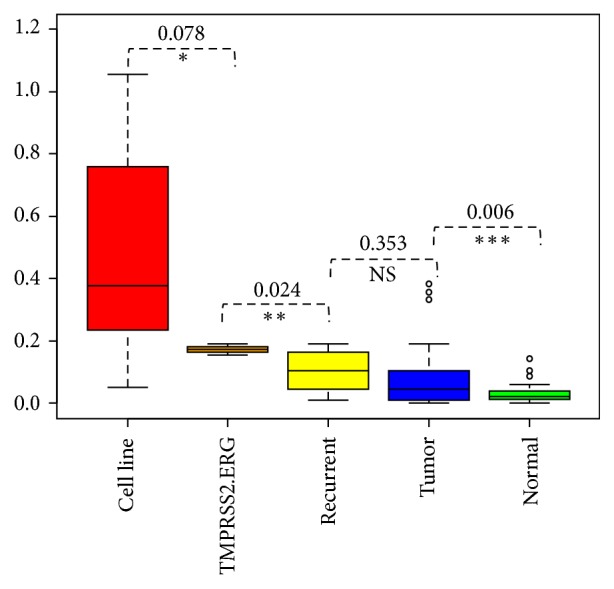
The boxplot comparison of iFCR^average^ values among four groups. The *x*-axis represented five different groups: C: cell lines group, TMPRSS2-ERG group, R: recurrent group, T: tumor groups, and N: normal tissue group. The *y*-axis is the iFCR value. The iFCR values in breast cancer cell lines are remarkably higher than other groups, and the iFCR values of tumor are remarkably higher than their normal counterparts. *T*-test was used to evaluate the statistical significance (*p* value) among different groups. NS is nonsignificance; ^*∗*^significance at 10% level, ^*∗∗*^significance at 5% level, and ^*∗∗∗*^significance at 1% level.

**Figure 5:**
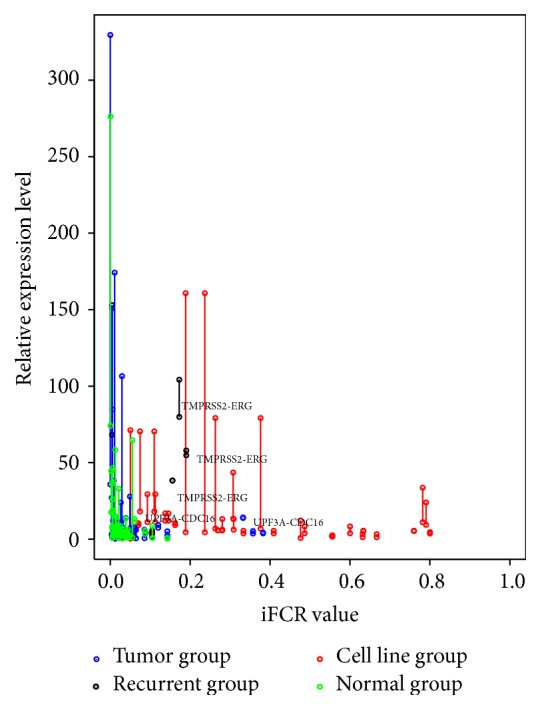
The relative RPKM expression level (*y*-axis) and iFCR value (*x*-axis) of parent's genes. Compared to primary tumors and normal tissues, fusion mutations occurring in breast cancer cell lines tend to be having higher iFCR value. The most frequent prostate cancer fusion mutation TMPRSS2-ERG appears as higher expression level and iFCR value in all three independent patients, and it is closed to the fusion mutation of cancer cell lines. A nonrecurrent tumor fusion mutation UPF3A-CDC16 from patient #9 is increased from 0.06 to 0.33 in the normal counterpart to its tumor sample.

The original prostate cancer study indicated [7] that the 14 prostate cancer samples harbored 38 tumor-specific chimeric transcripts, of which at least 5 are recurrent transcripts in Chinese population, including TMPRSS2-ERG, USP9Y-TTTY15, CTAGE5-KHDRBS3, RAD50-PDLIM4, and SDK1-AMACR. Specifically, the TMPRSS2-ERG is the most studied chimeric transcript in prostate cancer [17, 18]. As shown in Figure 4, we further divided the fusion mutations reported in primary prostate tumors into three groups based on their recurrence rates, which is the gold-standard for oncogenic potential evaluation in current studies [2, 7]. The first group consists of TMPRSS2-ERG, which was detected in three prostate cancer patients (i.e., TMPRSS2-ERG group). The second groups includes 5 recurrent fusion mutations reported in 8 prostate tumor samples (i.e., recurrent group). And the rest of tumor fusion mutations were included in the third group (i.e., tumor group). We also included fusion mutations reported in breast cancer cell lines (i.e., cell line group) and adjacent normal tissues as the “positive control” and “negative control,” respectively. After calculating iFCR for each group, we compared iFCR distribution across the different groups. Figures 4 and 5 showed iFCR^average^ values across five groups. Our results indicate that iFCR^average^ values are well correlated with recurrence rate of fusion mutation. As expected, the iFCR^average^ values are higher in breast cancer cell lines than those in other groups, and the iFCR^average^ values in adjacent normal tissues are the lowest. As the highest recurrent chimeric transcript, the group harboring TMPRSS2-ERG transcripts shows the highest iFCR^average^ values among primary prostate tumor groups. And then the iFCR^average^ values of the recurrent group are higher than the nonrecurrent group. The other two indicators, iFCR^max^ and iFCR^min^, show the same positive correlation trend with the recurrent rate of chimeric transcripts (see Supplement Figure 1 and Supplement Figure 2 in Supplementary Material available online at https://doi.org/10.1155/2017/9829175).

### 2.3. Novel Putative Oncogenic Fusion Mutations in the Prostate Cancer Dataset

The nonrecurrent fusion between exon 8 of ZC3H6 and exon 2 of LRP1B was present at a high iFCR value (0.38 for iFCR-average). The ZC3H6-LRP1B fusion was only detected in patient #13 and has not been previously reported, but its high iFCR value and the LRP1B did not seem to have any overlapping reads, indicating that it may play an important role in patient #13. The fusion mutation UPF3A-CDC16 was also identified in both the tumor and the adjacent normal tissue of the same patient (#9); did the iFCR value of this fusion mutation change between tumor and its adjacent normal tissue? We then compared the iFCR value of UPF3A-CDC16 in both tumor sample and its corresponding normal adjacent tissue. Interestingly, though it is a nonrecurrent chimeric transcript, the iFCR value of UPF3A-CDC16 was increased dramatically in tumor samples, from 0.06 in normal tissue to 0.33. This raises the possibility that this nonrecurrent chimeric transcript was under positive selection pressure and the clone harboring this specific transcript has been enriched during the progression of cancer in patient #9. However, more studies are required to clarify this mechanism for this observation.

## 3. Discussion

Since the discovery of gene fusion 50 years ago, over 358 oncogenic chimeric transcripts were recognized [3]. With advances in NGS and bioinformatics technology, identification of hybrid fusion gene is no longer a challenge. To date, one of the main challenges in gene fusion study is to help oncologists and physicians to identify oncogenic fusion genes from noisy “background” genomic aberrations.

It is widely accepted that subclone genetic heterogeneity is a common characteristic of tumors, with both spatial and temporal heterogeneity of primary tumors observed [11, 12]. The clonal evolution theory suggested that the survival ability of neoplastic cells could be inferred by comparing subclone diversity or architecture at different time points. Based on clonal evolution theory of cancer, a fusion gene is more likely to be a survival (oncogenic) aberration if the subclone harboring this specific mutation has larger clonal proportion in a heterogeneous tumor sample. And the subclone harboring specific “survival” genomic aberration could be positively selected and enriched during the cancer progression.

Traditionally, the subclone structure is recovered by in situ hybridization methods [13, 14] or computation methods based on DNA sequencing data [15, 16]. However, gene fusion studies often lack paired genome sequencing data. Here we proposed a novel method to estimate the subclone structure of fusion mutation based on transcriptome sequencing data only. We acknowledge that the assumption that expression in wild-type cells and tumor is similar might be flawed. However, our results suggested that iFCR could potentially reflect the tumor heterogeneity.

But the quantification of chimeric transcripts remains computationally challenging. Because the short sequencing reads from chimeric transcripts are almost the same as their parents' transcripts, it is very difficult to distinguish a chimeric transcript from their parents' transcripts. Current methods [19–26] identify chimeric transcripts by identification of fusion reads, that is (as shown in Figure 1), the short sequencing reads aligned onto the breakpoint of two parents' genes. These fusion reads are the only sequencing reads that can be used as evidence to support the occurrence of a certain chimeric transcript. In this work, we used the number of fusion reads to infer the expression level of chimeric transcripts and the number of overlapping reads to infer the expression level of parents' genes.

In this work, we tested our method on two public RNA-seq datasets and took a comparison of the chimeric subclone divergence among primary prostate tumors, normal prostate tissues, and breast cancer cell lines. As shown in Figure 1, we retrieved overlapping reads for parent genes through a realignment procedure. We compared the number of fusion reads and overlapping reads for fusion mutations among cancer cell lines, primary tumor samples, and normal tissues. The fusion mutations from primary tumors and normal tissues tended to have more overlapping reads (Figure 2). This is consistent with the fact that the somatic fusion mutations in primary tumor and normal tissue have relatively smaller subclone size than cancer cell lines, and the numbers of mutated clones might reflect the oncogenic potential of this fusion gene in a patient's tumor.

The nonrecurrent fusion between exon 8 of ZC3H6 and exon 2 of LRP1B was present at a high iFCR value (0.38 for iFCR-average). The ZC3H6-LRP1B fusion was only detected in patient #13 and has not been previously reported, but its high iFCR value and the LRP1B did not seem to have any overlapping reads, indicating that it may play an important role in patient #13. Previous studies have demonstrated that LRP1B is a potential tumor suppressor gene and downregulated expression of LRP1B proposed to be involved in multiple primary cancers [27, 28]. The deletion of LRP1B also has been associated with chemotherapy resistance in high-grade cancers [29]. These results indicate that the silencing of LRP1B may be a driver event.

Moreover, we found a very interesting fusion gene UPF3A-CDC16 in patient 9, whose iFCR value was increased from 0.06 to 0.33 in the tumor sample, compared with its adjacent normal tissue. This result indicated that UPF3A-CDC16 might be enriched during cancer progression. A previous study has suggested that* CDC16* is an important gene which involved cell reproduction [30]. One possible oncogenic mechanism is that the proportional increase of UPF3A-CDC16 might result in the function loss of CDC16, promoting the proliferation of neoplastic cells. Although it is possible that normal tissue had contaminated tumor samples during surgical operation or experimentation, the changes in iFCR values could still reflect its differential clone size.

Gene fusion events are not only a consequence of disability of cancer genomes, it is also an important mechanism of the evolution of novel proteins, it is contributing to the transcriptome complexity in normal tissues [31, 32]. Frenkel-Morgenstern and collaborators used mass spectrometry to study the corresponding protein products of chimeric transcripts and attempted to study potential functions of these chimeric products [33]. We hypothesized that the chimera products' new biological functions may rely heavily on their quantities. The relative expression level of chimeric transcripts might also be an indicator for inferring oncogenic potential of fusion mutation. Thus, we compared the iFCR value of chimeric transcripts and expression levels of their corresponding parents' genes. As Figure 5 shows, the expression levels of most of these genes are very low. However, the fusion mutations from breast cancer cell lines (red) exhibit higher iFCR value and were located at the right part of the diagram. The fusion mutations from tumor samples appear to have various iFCR values and, interestingly, the well-studied prostate cancer fusion TMPRSS2-ERG was closed to the fusions of cancer cell lines and appears to have higher iFCR values and expression levels in all three independent patients. Next, we calculated the fold change of parent genes' expression levels between tumor samples and their counterpart samples and compared the fold change with those fusions' iFCR values. As Supp. Figure 3 suggested, the fusion mutation of TMPRSS2-ERG changed the expression of its parent genes, indicating that the TMPRSS2-ERG mutation plays a critical role in prostate cancer dependent upon the expression changes of TMPRSS2 and ERG genes, consistent with previous widely discussed studies [34]. However, for the rest of high iFCR fusion mutations, such as ZC3H6-LRP1B, EMB-ATG10, UPF3A-CDC16, DYRK1A-CMTM4, and CD97-EMR2, the oncogenic potential remains unclear. The oncogenic mechanism of these chimeric transcripts might be different.

The advantage of our method is that the oncogenic potential of fusion genes could be estimated using a single RNA-seq dataset, which makes it ideal for application in precise medicine. Further works could integrate gene structural/functional information of fusion gene and our method to achieve better performance. The limitation of our method is difficult to evaluate its discriminative power by computational methods (e.g., cross-validation) due to the wide chimeric transcript spectrum among different tumor data. Also previous studies suggested that fusion genes were often caused by genomic segment amplifications, and these amplifications were often associated with gene overexpression [35].

In summary, we present a new concept of inferring the oncogenic potential of novel fusion genes identified in tumor samples. Unlike the existing structure/functional based method, our method incorporated the concept of clone evolution theory and transcription characterization of fusion genes. This study also showed that the iFCR values of fusion genes in tumor samples were remarkably higher than those in normal tissues, especially in tumor cell lines. The most frequent fusion mutation in prostate cancer TMPRSS2-ERG shows higher iFCR value in all three independent patients. We also observed that a previously reported [7] fusion gene, UPF3A-CDC16, was enriched in the tumor sample and it is indicated that UPF3A-CDC16 might be playing an important role during the cancer progression in patient 9#. To the best of our knowledge, this is the first work to incorporate transcriptome sequencing data and clone evolution theory to investigate the oncogenic potential of chimeric transcripts. Our work provides a new insight into the oncogenic potential study of fusion genes.

## 4. Methods

### 4.1. Data Source

A single-cell transcriptome sequencing study of glioblastoma (SRP042161) was used to test our RNA-seq data based on clone size estimation assumption. This dataset has 658 tumor single-cell sequencing libraries from five independent patients. They are MGH26 tumor sample with 189 single-cell sequencing libraries; MGH28 tumor sample with 95 single-cell sequencing libraries; MGH29 tumor sample with 96 single-cell sequencing libraries; MGH30 tumor sample with 91 single-cell sequencing libraries; MGH30L tumor sample with 91 single-cell sequencing libraries; and MGH31 tumor sample with 96 single-cell sequencing libraries.

The public RNA sequencing (RNA-seq) data of a prostate cancer study [7] (SRA: ERP000550) and a breast cancer study [19] (SRA: SRP003186) was downloaded from NCBI Sequence Read Archive (SRA) database.  Table 1 summarizes the datasets used in this study. The prostate cancer dataset was derived from 14 pairs of primary prostate cancer and their corresponding adjacent normal tissues in Chinese population. The breast cancer cell line dataset consists of 3 cell lines and 5 sequencing libraries; they are KPL-4, SK-BR-3 (two sequencing libraries), and BT-474 (two sequencing libraries). Since the MCF-7 cell line has not provided sequence of the chimeric transcripts, we excluded it from our analysis. The detailed descriptions of these datasets can be found in their original articles [7, 19]. In total, 28 paired-end RNA-seq libraries from the prostate cancer patients and 5 paired-end RNA-seq libraries from 3 distinct breast cancer cell lines were analyzed in this work.

### 4.2. Bioinformatics Preprocess Procedure

The fusion mutation detection procedure for single-cell sequencing libraries was conducted by FusionCatcher with default parameters [36], providing the BAM files and the information of sequencing reads which supported the chimeric transcripts. The iFCR^average^ values for single-cell libraries could be calculated by summing the reads supporting the fusions and their parents in these single-cells libraries from single patient. For each tumor sample, we also counted the number of cells each fusion was identified in and the number of cells that the parent genes in that fusion had nonzero transcript counts in and calculated a “real” ratio of the number of fusion clones and normal clones that is calculated from the cell counts rather than the transcripts counts.

For prostate cancer and breast cancer dataset, our focus was to predict the oncogenic potentials of chimeric transcripts. So, we directly used packages from previously published articles to detect fusion events and retrieve information for downstream analysis. For each sample, paired-end reads were aligned to their corresponding reference genome by a transcriptome aligner MapSplice [26] with default settings. As Figure 1 shows, the sequencing reads which span the breakpoints of parent genes were called “overlapping reads” and the sequencing reads spanning the breakpoint of chimeric transcript were called “fusion reads.” The overlapping reads were required to have at least 5 bp overlaps with flanking sequences in both sides of breakpoints. The number of fusion reads was directly obtained from their original publications [7, 19]. For breast cancer dataset, 24 validated fusion mutations were previously reported [19]. One fusion mutation (CSE1L-ENSG00000236127) was removed from our analysis due to the corresponding RefSeq gene symbol of ENSG00000236127 not being found in hg19. For the prostate cancer dataset, among 83 fusion mutations identified in their study [7], there are 4 (tumor samples) and 8 (adjacent normal tissues) fusion mutations that were removed in our further analysis due to the same reason. The detailed information of fusion mutations can be found in Supp. Table 1.

### 4.3. Internal Fusion Clone Ratio Calculation and Relevance Network Construction

In this work, we hypothesize that the ratio of the number of chimeric transcripts to the number of normal nonfusion transcripts could reflect the ratio of subclone population size. And this ratio could be estimated by the number of overlapped reads and fusion reads. The proposed subclone ratio estimator is defined as

(1) iFCRaverage=fa,bavgna,nbiFCRmax=fa,bmin⁡na,nbiFCRmin=fa,bmax⁡na,nb.

Here, *f*_*a*,*b*_ is the number of the fusion reads mapping to the breakpoint of gene *a* and gene *b*. *n*_*a*_ is the number of overlapping reads for gene *a* and *n*_*b*_ is the number of overlapping reads for gene *b*. Here avg(*n*_*a*_, *n*_*b*_), min⁡(*n*_*a*_, *n*_*b*_), and max⁡(*n*_*a*_, *n*_*b*_) donate the relative expression of wild-type transcript from parent genes *a* and *b* using three simple combinations. And thus iFCR^average^, iFCR^max^, and iFCR^min^ represent the average, maximum, and minimum ratio of chimeric transcripts subclones to wild-type subclones, respectively. This equation could be refined later with the number of reads replaced by RPKM (the number of reads per kilobase of gene length per million mappable reads) [7, 37].

## Supplementary Material

Figure S1: The boxplot comparison of iFCR^max^ values among four groups.
Figure S2: The boxplot comparison of iFCR^min^ values among four groups.
Figure S3: The comparison of fold changes of parents' genes and iFCR value (*x*-axis).

## Figures and Tables

**Table 1:** Summary of three validated datasets used in this study.

	Sample type	Sequencing libraries	Chimeric transcripts
^#^Single cell	Single cell	658	574

Prostate cancer, 14 individuals	Tumor samples	14	40
	Adjacent normal tissue	14	37

Breast cancer cell lines	BT-474	2	9
	KPL-4	1	3
	SK-BR-3	2	9

^#^The total number of fusion mutations in single cell dataset.
