# Supplementary material for: RNA-seq Based Transcription Characterization of Fusion Breakpoints as a Potential Estimator for Its Oncogenic Potential
Source: Biomed Res Int. 2017 Oct 17;2017:9829175. doi: 10.1155/2017/9829175 (PMC5664375; doi:10.1155/2017/9829175)
Supplement: Supplementary file 1 — Figure S1: The boxplot comparison of iFCRmax values among four groups. Figure S2: The boxplot comparison of iFCRmin values among four groups. Figure S3: The comparison of fold changes of parents' genes and iFCR value (x-axis). [file 9829175.f1.zip › Supp Figures.docx]

# Supplementary Material

# RNAseq based transcription characterization of fusion breakpoints as a potential estimator for its oncogenic potential

## Supp Fig 1. The boxplot comparison of iFCRmax values among four groups. The x-axis represented the five different groups: C: Cell Lines; TMPRSS2-ERG group, R: recurrent group, T: tumor groups and N: normal group. The y-axis is the iFCRmax value. The iFCRmax values in breast cancer cell lines are remarkably higher than prostate cancer groups, and the iFCRmax values of tumor mutations are remarkably higher than their normal counterparts.

## Supp Fig 2. The boxplot comparison of iFCRmin values among four groups. The x-axis represented five different groups: C: Cell Lines; TMPRSS2-ERG group, R: recurrent group, T: tumor groups and N: normal group. The y-axis is the iFCRmin value. The iFCRmin values in breast cancer cell lines are remarkably higher than prostate cancer groups, and the iFCRmin values of tumor mutations are remarkably higher than their normal counterparts.


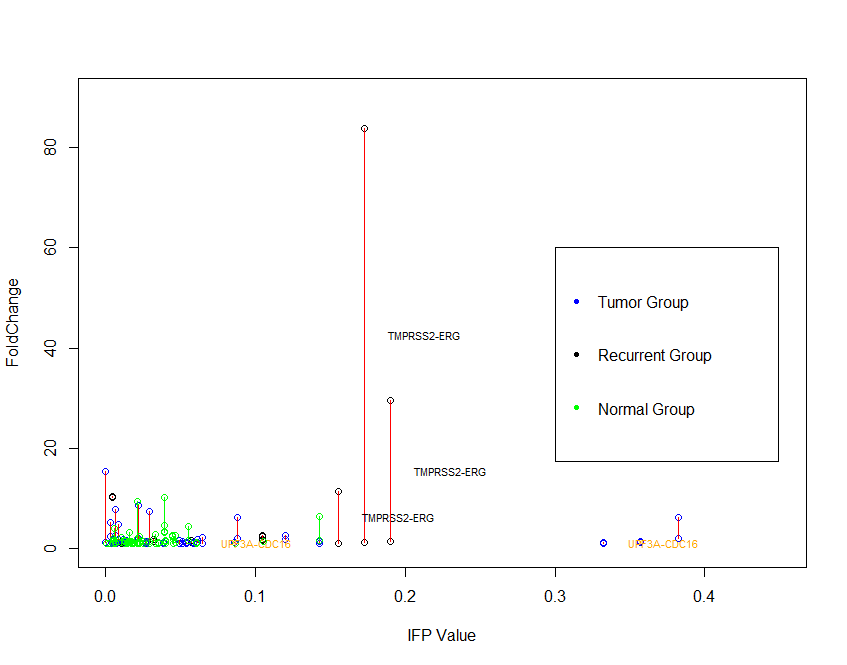


## Supp Fig 3. The comparison of fold changes of parents’ genes and iFCR value (x-axis). X-axis represents iFCR value, y-axis is fusion parents’ expression fold change between tumor and normal counterpart samples. The TMPRSS2-ERG might have changed the expression level of parent genes.
